# Physical activity and sedentary behaviour of primary school learners in the Eastern Cape province of South Africa

**DOI:** 10.4102/safp.v64i1.5381

**Published:** 2022-03-22

**Authors:** Howard Gomwe, Eunice Seekoe, Philemon Lyoka, Chioneso S. Marange, Dennyford Mafa

**Affiliations:** 1 Department of Teaching, Learning and Community Engagement, Faculty of Health Sciences, Sefako Makgatho Health Sciences University, Medunsa, South Africa; 2 Department of Nursing Science, Faculty of Health Science, University of Fort Hare, East London, South Africa; 3 Department of Statistics, Faculty of Science, University of Fort Hare, East London, South Africa; 4 Department of Social Science, Faculty of Humanities, University of Fort Hare, Alice, South Africa

**Keywords:** children, learners, physical activity, sedentary behaviour, South Africa

## Abstract

**Background:**

This study was designed to assess physical activity (PA) levels and sedentary behaviour amongst primary school learners in the Eastern Cape province of South Africa.

**Methods:**

A cross-sectional study was adopted to assess the patterns and levels of PA and sedentary behaviour using the Physical Activity Questionnaire for Older Children (PAQ-C). The sample consisted of primary school learners, both boys and girls, aged 9–14 years. The learners were randomly selected from rural, urban and peri-urban areas in the Eastern Cape province of South Africa.

**Results:**

Using a complete case analysis, 870 randomly selected participants (boys = 351 and girls = 519) aged 9–14 years were retained. Overall, the sample had a low mean PAQ-C score of 2.33 ± 0.43. The mean of PA in boys was significantly higher (*p* = 0.003) in comparison with the girls. The 13- to 14-age group had significantly higher PA levels (*p* = 0.014). Learners from urban areas (*n* = 136; 77.3%) engaged more in sedentary behaviour compared to those from rural areas (*n* = 252; 54.9%).

**Conclusion:**

The findings demonstrated low levels of PA and high engagement of sedentary behaviour across the combined gender groups, which have negative implications on health, growth and development of children. The study, therefore, recommends relevant stakeholders to implement interventions aimed at promoting an increase in PA and a reduction in sedentary behaviours for primary school learners in the Eastern Cape province of South Africa.

## Introduction

South Africa just like any other country is not immune to the issues of addressing challenges related to physical inactivity and sedentary lifestyle amongst primary school learners. Studies have increasingly shown an inverse relationship between physical activity (PA) levels and time spent in sedentary behaviour.^1^ Assessing PA and sedentary behaviour of school learners is vital for intervention purposes.^2^ Physical activity is defined as any bodily movement produced by skeletal muscles that require use of energy as well as any kind of movement during leisure time, walking to and from places or as part of a person’s daily routine work.^3^ Physical activity may be planned or unplanned, and the resultant is energy expenditure, as well as improvement of physical fitness.^3^ It involves all types of moderate-to-vigorous body movements that include energy expenditure and encompasses a wide range of formal and informal activities.^4^ Sedentary behaviour, however, is considered to be a group of habits that usually happen as an individual sits or lies, where little energy is required.^5,6^ Such behaviour has been described as a cardiovascular risk by some researchers. Regular participation in moderate- to high-intensity PA has been proved to protect individuals from chronic diseases and conditions such as diabetes, obesity and coronary heart diseases.^7,8,9^ Understanding the patterns and levels of PA and sedentary behaviour is important for intervention purposes as well as to encourage lifestyles that are more active in order to prevent chronic diseases.^10,11^ In the literature, demographics like age, gender and residence are reported to be possible influential factors of PA levels and sedentary behaviour.^12^

According to the Centres for Disease Control and Prevention (CDC),^13^ recommended levels of PA for children and adolescents 6–17 years include a minimum of at least 60 min daily PA of moderate-to-high intensity.^14^ The PA levels must furthermore conform to recommendations regarding variation, enjoyment and the developmental level of the learner.^13^ Recommended maximum time spent on sedentary activities for learners is 2 h per day with reference to screen time, which thus entails time spent watching television or using the computer, a cell phone or a tablet, amongst others.^15^ The decline in PA levels and increasing sedentary behaviour amongst school learners in many countries including South Africa could be because of many factors related to perceived deficiencies in the curriculum material and socio-economic factors like culture, gender, time, lack of facilities and human and financial resources.^16^ Within the South African basic education system, for example, life orientation (LO) has replaced physical education (PE).^17^ A study done on teachers’ perspectives on the implementation of this new subject showed that 36% of the LO teachers are not qualified to teach PE.^17^

It has been noted that, based on the South African perspective, there has been a gradual decline in both school learners’ physical fitness levels and activity levels in primary school learners.^18^ This is mainly attributed to the easy and necessary access to transport as the unsafe environment, and the distances between schools and residential areas make it difficult to walk to and from school.^16,19^ A study carried out in South Africa^19^ tested 767 boys and girls (age range 9–15 years old) to determine the time they spend on PA. The results showed that a decrease in PA was associated with age and an increase in sedentary behaviour. Moreover, a study by Craig et al.^20^ showed that only 26% of school learners in the rural areas of South Africa meet the recommended PA levels. Although rural school learners walk or travel long distances on foot, the majority still do not meet the recommended PA levels.^20^ Findings from a study conducted in Tlokwe Local Municipality, South Africa by Toriola and Monyeki,^1^ revealed that there are declining levels of PA amongst school learners where, out of 283 learners, 39% of boys and 16% of girls were insufficiently active. Furthermore, Micklesfield et al.^21^ assessed PA levels amongst primary school learners in Mpumalanga province of South Africa and showed declining levels of PA that have affected physical fitness levels of learners.

Results of research studies in other countries show similar and somewhat conflicting findings on primary school learners’ PA levels and sedentary behaviour. Koorts et al.^22^ assessed the types and PA levels of adolescents in South-West of England for 2728 adolescents (1299 boys and 1429 girls). The results revealed that the boys complied better with the international guidelines than the girls. Boys were highly active outside school. They participated in more sports activities on school days and in more community sports activities on non-school days than girls. In Finland, Hartikainen et al.^23^ evaluated the PA and sedentary behaviours of school children (third and fifth grades) during school hours. The research was carried out just before and after the curriculum reforms of Finnish basic education in 2014. Results showed significant differences in PA levels before and after the reforms with a greater proportion of moderate-to-vigorous intensity PA (MVPA) after the reforms. In Munich Germany, Kuritz et al.^24^ investigated the PA and sedentary behaviour of 322 school children (198 full-day learners and 134 half-day learners). The results showed that children who were attending full day showed the highest percentage of MVPA (13.7%) and the lowest percentage of sedentary behaviour (49.5%). Kidokoro et al.^25^ evaluated the PA and sedentary behaviour levels of primary school children aged between 9 and 12 years of both boys and girls from Kenya (*n* = 122) and Japan (*n* = 176). The results indicated that Kenyan children spent more time in MVPA compared to Japanese children who used active transport.

Wachira^26^ assessed PA and sedentarism levels of the Kenyan school children in Nairobi. From 563 children (boys and girls aged 9–11 years), the researcher reported that 14.3% of the children were sufficiently active with more boys (20.2%) meeting the recommended PA levels than girls (9.3%). Overall, 15.5% of the children had high sedentarism and spent more than 4.25 h in screen-based sedentary activities on weekends. Gerber et al.^12^ compared the PA levels and sedentary behaviour of primary school children (boys and girls) in Côte d’Ivoire (*n* = 499), South Africa (*n* = 1074) and Tanzania (*n* = 395). The results showed that most children met WHO recommendations for MVPA: 89.6% (boys: 91.7%, girls: 87.4%) in Côte d’Ivoire, 76.9% (boys: 91.0%, girls: 62.4%) in South Africa and 93.8% (boys: 95.5%, girls: 92.0%) in Tanzania. Girls were found to be engaged in more sedentary behaviour. Both sedentary behaviour and MVPA were higher amongst older school children as compared to their younger peers. In a study by LeBlanc et al.,^27^ findings revealed that 54% of the learners did not meet the international guidelines for screen time. According to the South African Youth Risk Behaviour Survey,^28^ less than 43% of the school learners met the recommended levels. The survey by the South African Youth Risk Behaviour shows significant differences in participation levels between boys and girls, where more boys participate in PA as compared to girls.^22^ The reason is that to a greater extent, girls show somewhat negative attitudes towards PA, and overall, there is a decline in PA participation levels of school learners.^29^ The aim of this study was to evaluate the PA levels and sedentary behaviour of primary school learners in the Eastern Cape province of South Africa.

## Materials and methods

### Design of the study

The study adopted a quantitative approach and made use of a cross-sectional study to assess the patterns and levels of PA and sedentary behaviour of primary school learners in the Eastern Cape province, South Africa.

### Study setting, sampling and sample size determination

The Eastern Cape province is situated on the eastern seaside of South Africa. It is the second largest province after the Northern Cape. The population is dominated by the Xhosa-speaking people who mainly depend on government grants and subsistence farming. The province has six districts, namely, OR Thambo, Chris Hani, Alfred Nzo, Sarah Baartman, Joe Gqabi and Amathole and two metropolitan municipalities, namely, Nelson Mandela Bay and Buffalo City. The research study was conducted in Buffalo City Metropolitan (Amathole municipality), Oliver Tambo and Chris Hani municipalities. These three municipalities represent urban, peri-urban and rural communities and were conveniently and purposively selected for the purposes of comparison of the diverse contexts that exist in the targeted geographical spaces. From these settings, it is possible to get a comprehensive picture of the school learners’ PA participation patterns in the Eastern Cape province. There are 5589 public schools, both primary and secondary, in the province and the total population of learners is 1 157 901, with 633 910 in primary schools.^30^ The district education department of each selected municipality provided a list of schools from quintiles 1, 2 and 3 on which random selection of schools was based. The quintile ranking is a system that the South African Department of Education uses to divide all public schools for purposes of allocation of financial resources. In quintile one, there are schools that are the poorest, whilst in quintile five, there are schools that are least poor. We considered schools only in quintiles 1, 2 and 3 that are declared to be no-fee paying schools, whilst those in quintiles 4 and 5 are fee-paying schools. Overall, 18 primary schools from quintiles 1, 2 and 3 were randomly selected using a computer-generated programme. Thus, six schools were randomly selected from Amathole municipality (three urban and three peri-urban schools), whilst 12 schools were randomly selected from Oliver Tambo (six schools) and Chris Hani (six schools) municipalities (rural setting). Lastly, class registers were used to randomly draw a 10th of the population (learners who met the inclusion criteria) from each randomly selected school. The exclusion criteria included learners with disability (because of the strenuous activities for assessing PA), learners less than 9 years old and also learners who were more than 14 years old. A total of 876 participants who met the inclusion criteria were recruited and participated in the study.

### Data collection instrument

We used the Physical Activity Questionnaire for Older Children (PAQ-C), which has been previously validated in an ethnically diverse and similar cohort of South African children.^31,32^ In addition, the PAQ-C has demonstrated a good internal consistency^33^ and acceptable test-retest reliabilities.^34^ Convergent and construct validity of the PAQ-C has also been established, with moderate associations found with the activity rating scale.^35^ The questionnaire measures general PA levels in children aged 8–14 years during a typical week in a school year. The questionnaire was used to gather information pertaining to the child’s participation in a broad spectrum of different physical (in)activities across several domains. These domains included school PE, informal activities, sedentary activities after school, transport to and from school and some extramural activities. The questionnaire was piloted on a convenient sample of children from the same age group and similar demographic profile prior to the main data collection. This sample did not form part of the final analysis. The primary purpose of the pilot testing was to ensure clarity of the terminology used and the applicability and availability of the listed sport activities. Modifications to certain parts of the questionnaire were performed after the pilot study; for example, cross-country skiing, ice hockey and badminton were removed and replaced with soccer, athletics and rugby to suit the local context. A final mean score categorised learners as having low, moderate or high PA levels. Low levels of PA were 1.00–2.33, moderate levels 2.34–3.66 and high levels 3.67–5.00.^36^

Sedentary behaviour was measured by a modified self-administered PA checklist (SAPAC) indicating time spent on sedentary activity.^37^ The learners were asked what sedentary activities they were engaged in during the past 7 days of the typical week. They were asked to indicate time spent (coded as 0 = None; 1 = Less than an hour; 2 = 1 h; 3 = 2 h; 4 = 3 h or more) on 11 sedentary behaviour activities, which include time spent on computers or the Internet, sitting, playing video games, reading books, listening to music, television and movies. This measure showed acceptable levels of test–retest reliability and validity in other similar studies.^37^ We used a 2 h per day cut off for sedentary activities based on the recommendation of the American Academy of Paediatrics that children should engage in sedentary activities for less than 2 h each day.^38^ Demographic features that included gender, age and geographical location were also added to the questionnaire.

### Data analysis

Statistical Package for Social Sciences (SPSS) version 27 was used for data analysis. We opted for a complete case analysis by only including participants with no missing data on the variables of interest. From the 876 respondents, a total of six participants with missing data were excluded from the analysis. A descriptive approach was used to analyse the study’s demographic features, as well as to examine the patterns and mean levels of sedentary behaviour and PA of the sampled learners. For mean comparisons of the established mean levels of PA on gender, a non-parametric Mann Whitney *U* test was used because of the non-normality of the mean level. Lastly, a Kruskal–Wallis H test coupled with its post-hoc pair-wise comparison tests was used to compare the mean levels of PA across age groups and residence.

### Ethical considerations

The study involved primary school learners, both boys and girls aged 9–14 years old, who were randomly selected from rural, urban and peri-urban areas in the Eastern Cape province of South Africa. The research ethics committee of the University of Fort Hare approved the study protocol and issued ethical clearance (certificate reference number: LYO011SGOM01), giving permission for the study to be conducted. Moreover, the researchers sort permission to conduct the study from the research ethics committees of the Department of Education as well as the Department of Health in the Eastern Cape province of South Africa. After obtaining all the necessary ethical clearance letters, the researchers then initiated the data collection process. The scope and nature of the study were explained to the selected primary school learners as well as their parents. As the study was looking at learners aged 9–14 years, parental consent was obtained. Confidentiality and anonymity were maintained as guided by the study protocol.

## Results

The resultant sample consisted of 351 (40.34%) boys and 519 (59.66%) girls. A total of 222 (25.52%) were in the age group 9–10 years, whereas most of them (*n* = 423; 48.62%) were in the age group 11–12 years. The age group 13–14 years had 225 participants. The majority resided in rural areas (52.76%; *n* = 459), whilst 20.23% (*n* = 176) and 27.01% (*n* = 235) resided in urban and peri-urban areas, respectively. Overall, the sample had a PAQ-C score of 2.33 ± 0.43 for PA (see Table 1). According to Kowalski et al.,^36^ the overall PAQ-C score of 2.33 is regarded as low but on the high end of low PA levels. However, the mean level of PA in boys (mean = 2.39; s.d. = 0.44; *n* = 351), which, according to Kowalski et al.^36^ is regarded as moderate, was significantly higher (*p* = 0.003) in comparison with the girls (mean = 2.29; s.d. = 0.42; *n* = 519), which is regarded as low. Thus, boys had moderate PA levels (on the low end of moderate PA levels), whilst girls had low PA levels (on the high end of low PA levels).

**TABLE 1a:** Summary levels, frequencies and percentages of physical activity (PA) and sedentary behaviour classifications by gender.

By Gender	Combined (*N* = 870)			Boys (*N* = 351)			Girls (*N* = 519)			*p*-value
	Mean ± s.d.	*n*	%	Mean ± s.d.	*n*	%	Mean ± s.d.	*n*	%	
**Mean PA levels (PAQ-C Score)**										
Combined	2.33 ± 0.43	-	-	2.39 ± 0.44	-	-	2.29 ± 0.42	-	-	0.003*
**PA levels (PAQ-C Score) frequency and percentage distribution**										
Low	-	464	53.3	-	171	48.7	-	293	56.5	-
Moderate	-	404	46.4	-	179	51.0	-	225	43.4	-
High	-	2	0.2	-	1	0.3	-	1	0.2	-
**Sedentary behaviour per day (hours) frequency and percentage distribution**										
< 2 hours per day	-	333	38.3	-	142	40.5	-	191	36.8	-
≥ 2 hours per day	-	537	61.7	-	209	59.5	-	328	63.2	-

* , Statistically significant differences (alpha = 0.05).

**TABLE 1b:** Summary levels, frequencies and percentages of physical activity (PA) and sedentary behaviour classifications by age.

By Age	9–10 years (*N* = 222)			11–12 years (*N* = 423)			13–14 years (*N*= 225)			*p*-value
	Mean ± s.d.	*n*	%	Mean ± s.d.	*n*	%	Mean ± s.d.	*n*	%	
**Mean PA levels (PAQ-C Score)**										
Combined	2.32‡ ± 0.49	222	-	2.30‡ ± 0.41	423	-	2.40† ± 0.41	225	-	0.014*
Boys	2.40 ± 0.56	76	-	2.36 ± 0.39	164	-	2.43 ± 0.43	111	-	0.302
Girls	2.28 ± 0.44	146	-	2.26 ± 0.42	259	-	2.37 ± 0.39	114	-	0.093
**PA levels (PAQ-C Score) frequency and percentage distribution**										
Low	-	121	54.5	-	239	56.5	-	104	46.2	-
Moderate	-	100	45.0	-	183	43.3	-	121	53.8	-
High	-	1	0.5	-	1	0.2	-	0	0.0	-
**Sedentary behaviour per day (hours) frequency and percentage distribution**										
< 2 hours per day	-	80	36.0	-	154	36.4	-	99	44.0	-
≥ 2 hours per day	-	142	64.0	-	269	63.6	-	126	56.0	-

* , Statistically significant differences (alpha = 0.05).

†, ‡ , grouping for the Kruskal-Wallis post-hoc pairwise comparisons, where (†, ‡) represents statistically significant different mean levels using asymptotic significances (2-sided tests) adjusted by the Bonferroni correction for multiple tests.

**TABLE 1c:** Summary levels, frequencies and percentages of physical activity (PA) and sedentary behaviour classifications by residence.

By Residence	Urban (*N* = 176)			Peri-Urban (*N* = 235)			Rural (*N* = 459)			*p*-value
	Mean ± s.d.	*n*	%	Mean ± s.d.	*n*	%	Mean ± s.d.	*n*	%	
**Mean PA levels (PAQ-C Score)**										
Combined	2.13‡ ± 0.34	176	-	2.15‡ ± 0.37	235	-	2.50† ± 0.42	459	-	< 0.0001*
Boys	2.14‡ ± 0.32	53	-	2.16‡ ± 0.43	104	-	2.59† ± 0.38	194	-	< 0.0001*
Girls	2.12‡ ± 0.35	123	-	2.14‡ ± 0.31	131	-	2.44† ± 0.44	265	-	< 0.0001*
**PA levels (PAQ-C Score) frequency and percentage distribution**										
Low	-	126	71.6	-	180	76.6	-	158	34.4	-
Moderate	-	50	28.4	-	54	23.0	-	300	65.4	-
High	-	0	0.0	-	1	0.4	-	1	0.2	-
**Sedentary behaviour per day (hours) frequency and percentage distribution**										
< 2 hours per day	-	40	22.7	-	86	36.6	-	207	45.1	-
≥ 2 hours per day	-	136	77.3	-	149	63.4	-	252	54.9	-

* , Statistically significant differences (alpha = 0.05).

†, ‡ , grouping for the Kruskal-Wallis post-hoc pairwise comparisons, where (†, ‡) represents statistically significant different mean levels using asymptotic significances (2-sided tests) adjusted by the Bonferroni correction for multiple tests.

**FIGURE 1:**
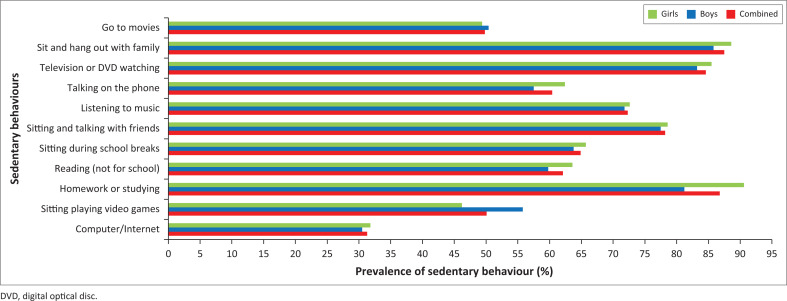
Types of sedentary behaviour.

Figure 1 shows that the sedentary activities in which the total group participated most include being busy doing homework and studying (86.8%), sitting and talking with friends (78.2%), listening to music (72.3%), watching television or digital optical discs (DVDs) (84.6%) and sitting and hanging out with family/relatives (87.5%). The two sedentary activities in which boys demonstrated the higher participation than girls were sitting playing video games (55.8% vs 46.2%) and going to movies (50.4% vs. 49.4%). However, it should be noted that the gender difference on ‘going to movies’ is negligible. Generally, girls participated the most in the majority of the sedentary behaviours, that is, sitting and hanging out with family/relatives, watching television or DVDs, talking on the phone, listening to music, sitting and talking to friends, sitting during school breaks, reading (not for school), doing homework or studying and browsing the Internet.

Table 1 also shows the frequencies and percentages of sedentary behaviour and PA classifications with gender. The majority of the learners (*n* = 537; 61.7%) engage in a sedentary lifestyle, most of whom are girls (*n* = 328; 63.2%). This result was consistent across the different gender groups with the majority of both boys (*n* = 209; 59.5%) and girls (*n* = 328; 63.2%) reported to be having sedentary behaviour. In terms of PA by gender, the majority of the learners (as combined) reported low PA levels (*n* = 464; 53.3%) with 404 (46.4%) having moderate levels of PA, whilst only two (0.2%) had high levels of PA. Similarly, the majority of girls engage in low levels of PA (*n* = 293; 56.5%), whilst only 0.2% (*n* = 1) reported high PA levels.

In Table 1, we also have the levels of PA by the respective age groups of participants. Results show that higher levels of PA were observed in learners in the age group 13–14 years (mean = 2.40; s.d. = 0.41; *n* = 225) as compared to other age groups. Learners in the age group 11–12 years had the lowest levels of PA (mean = 2.30; s.d. = 0.41; *n* = 423). Age groups in both boys and girls reported a similar distribution in patterns of PAQ-C scores. Boys had higher levels of PA as compared to the girls across all the different age groups. The Kruskal–Wallis H test revealed a statistically significant difference (*p* = 0.014) on the PAQ-C scores for the different age groups on the combined gender group. Using the Kruskal–Wallis post-hoc pairwise comparisons, the differences were statistically significant between the combined mean scores of the 9- to 10- and 13- to 14-year-olds as well as the 11- to 12- and 13- to 14-year-olds, whereas the differences were not significant between the 9- to 10- and 11- to 12-year-olds. Thus, the results suggest that the 13–14 age group had a significantly higher PA level than the other age groups. In general, the 13- to 14-year-olds as well as all boys across all age groups had moderate PA, whilst all girls from 9 to 12 years of age had low PA levels.

The frequencies and percentages of sedentary behaviour and PA classifications with age (see Table 1) revealed that the majority in all the various age groups engage in sedentary behaviour. In terms of PA, the majority of 9- to 10-year-olds (*n* = 121; 54.5%) as well as 11- to 12-year-olds (*n* = 239; 56.5%) reported low levels of PA. As for the 13–14 years age group, the majority (*n* = 121; 53.8%) reported to be engaging in moderate PA with 104 (46.2%) reporting low levels of PA engagement.

The patterns of PA levels with residence (see Table 1) reveal that learners from rural areas had higher levels of PAQ-C scores (mean = 2.50; s.d. = 0.42; *n* = 459) as compared to those from peri-urban (mean = 2.15; s.d. = 0.37; *n* = 235) and urban areas (mean = 2.13; s.d. = 0.34; *n* = 176). A similar pattern in the distribution of PA levels across residence was also reported in boys-only and girls-only groups. The Kruskal–Wallis H test revealed statistically significant differences in the levels of PA for the combined (*p* ≤ 0.0001), boys-only (*p* ≤ 0.0001) and girls-only (*p* ≤ 0.0001) groups. The Kruskal–Wallis post-hoc pairwise comparisons also showed that for the categories combined, boys-only and girls-only groups, the levels of PA of rural learners are significantly higher than that of peri-urban and urban learners. However, the Kruskal–Wallis post-hoc pairwise comparisons showed that the levels of PA were not significantly different for peri-urban and urban learners. In general, the results show that learners from the rural areas had moderate levels of PA, whilst those from urban and peri-urban areas had low levels of PA.

Accessing the frequencies and percentages of sedentary behaviour and PA classifications with residence, the majority of urban (*n* = 136; 77.3%), peri-urban (*n* = 63.4%) and rural (*n* = 252; 54.9%) learners engage in a sedentary lifestyle (see Table 1). In terms of PA, the majority of urban learners (*n* = 126; 71.6%) as well as peri-urban learners (*n* = 180; 76.6%) reported low levels of PA. In contrast, the majority of rural learners (*n* = 300; 65.4%) reported to be engaging in moderate PA with 158 rural learners (34.4%) reporting low levels of PA engagement.

## Discussion

This study provides an assessment of PA levels and sedentary behaviour patterns for urban, peri-urban and rural primary school learners in South Africa. From this study, it is clear that the majority (53.3%) of the participants engage in low PA participation. Thus, generally, the sampled primary school children do not comply with the health-based guidelines for PA and do not meet the recommended levels of daily PA endorsed by the World Health Organization.^10,17^ On the contrary, Gerber et al.^12^ reported that most primary school children (76.9%) in South Africa met WHO recommendations. In PA participation, we found out that the majority of the learners reported low PA levels (PAQ-C score of 2.33 ± 0.43), especially girls. Thus, the majority of learners engage in low PA (*n* = 464; 53.3%) participation. However, the majority of boys engage in moderate PA (*n* = 179; 51.0%) as compared to girls (*n* = 225; 43.4%). Thus, generally, the results show that boys are more physically active than girls, a trend generally reported in the literature,^18,22^ and studies in South Africa also reported similar results.^15,19,21^ This might be explained by the fact that boys prefer outdoor games or competitive games, whilst girls prefer indoor activities or concentrate on household chores.^20^ Our findings also revealed that PA participation increases with age and these findings have been reported in the literature.^19,12^ The majority (*n* = 121; 53.8%) of the 13- to 14-year-olds engage in moderate PA participation, whereas the majority of 9- to 10-year learners (*n* = 121; 54.5%) and 11- to 12-year (*n* = 239; 56.5%) learners engage in low PA participation. Our findings contradict those of McVeigh and Meiring,^19^ who reported that in a cohort of South African children aged between 9 and 15 years, there was a decrease in PA participation with age.

Regarding the level of PA participation according to the residence, rural learners reported to be more engaged in PA compared to the urban and peri-urban learners. The majority of learners from rural areas (*n* = 300; 65.4%) participated in moderate PA compared to the majority of those in urban areas (*n* = 126; 71.6%) and peri-urban areas (*n* = 180; 76.6%) who were involved in low PA participation. Thus, from our study, rural learners are the only group of learners that met the recommended PA participation levels. This can be attributed to the differences in lifestyle within these different settings. In rural areas, most learners tend to walk long distances and engage in more physical household chores compared to those in the urban and peri-urban settings. Our results for rural learners contradict that of Craig et al.^20^ who reported that only 26% of school learners in the rural areas of South Africa meet the recommended PA levels and further elaborated that although rural school learners walk long distances on foot, the majority still do not meet the recommended PA levels.^20^ In contrast, LO that replaced PE is not empowering enough skills to school learners to participate in physical activities. It would be ideal for the Department of Education in South Africa to revise its LO curriculum and try to adopt well-tested curricula from other countries. For example, in Finland, Hartikainen et al.^23^ reported significant differences in PA participation levels before and after curriculum reforms with a greater proportion of MVPA after the reforms. It is widely believed that declining levels of PA have affected physical fitness levels of learners over time, and this decline has significantly contributed towards several health risks like hypertension, obesity, diabetes mellitus and chronic heart diseases.^21^ Literature suggests that learners from both high-income and low-to middle-income countries do not meet the global PA standards.^17^

With regard to sedentary activities, the average total time of the combined group was more than 2 h per day (2.33 ± 0.43). This result was consistent across the different gender groups with the majority of both boys (59.5%) and girls (63.2%) reported to be having sedentary behaviour. The results of this research study are similar to those of Uys^17^ who reported a rising of sedentary lifestyle amongst primary school learners and that of McVeigh and Meiring,^19^ whose results showed that the majority of both boys and girls reported to be having sedentary behaviour. Moreover, 61.7% of all learners engage in a sedentary lifestyle of which the majority are girls (61.0%). Our findings are supported by those of Gerber et al.^12^ who reported that girls were engaged in more sedentary behaviour than boys. Sedentary behaviour classification with age revealed that the majority of all the various age groups engage in sedentary behaviour. However, learners in the 9–10 years age group (*n* = 142; 64.0%) had the highest levels of sedentary behaviour, whilst the lowest levels of sedentary behaviour were recorded in the 13–14 years age group (*n* = 126; 56.0%). This was a similar distribution in patterns of sedentary behaviour levels by age groups in boys and girls separately. This shows that sedentary behaviour decreases by age. Our results are similar to the findings of Craig et al.^20^ that a decrease in sedentary behaviour was associated with an increase in age. However, on the contrary, McVeigh and Meiring^19^ reported an increase in sedentary behaviour with age. Sedentary behaviour was also reported to be higher amongst older school children as compared to the younger children.^12^ The patterns of sedentary behaviour with residence indicated that learners from urban (*n* = 136; 77.3%) and peri-urban areas (*n* = 149; 63.4%) had higher levels of sedentary behaviour as compared to those from rural areas (*n* = 252; 54.9%). This is mainly attributed to the easy access to transport because of the unsafe environment, and the distance between schools and residential areas makes it difficult for these learners to walk to school.^16,17^ The major sedentary activities in this study are watching TV, sitting and hanging around with friends or family, doing homework or studying, listening to music, playing on a computer or using the Internet and watching movies. The majority of the learners are not meeting the guidelines of spending 2 h only on sedentary activities per day according to WHO.^10,12,14,26^ Leading a healthy lifestyle seems to be an expensive undertaking amongst communities of South Africa, including school learners. Childhood obesity or being overweight is currently one of the major challenges amongst school learners in most parts of South Africa.^18^ One of the reasons for the low levels of PA, as well as increasing levels of sedentary behaviour amongst the learners in this study, can be deficiencies in the curriculum, socio-economic factors as well as lack of human and financial resources.^16^

## Conclusion

The majority of primary school learners in the Eastern Cape province of South Africa engage in low PA participation, especially girls. Older age groups are spending the least time performing sedentary tasks compared to younger age groups. These behaviour patterns correlated with their levels of PA being the highest of all the age groups. The findings of this study indicate that the PA levels and sedentary behaviour of primary school learners of the Eastern Cape province of South Africa do not meet with the health-based guidelines for PA and sedentary behaviour. Sedentary behaviour seems to be replacing PA in primary school learners. The study, therefore, recommends relevant stakeholders to implement targeted theory-based, contextually appropriate interventions,^39,40^ which are aimed to promote physical activities and reduce sedentary behaviours in primary school learners in the Eastern Cape province of South Africa. Thus, children with low fitness could derive health benefits by improving their PA level as this is associated with numerous physiological and psychological health benefits.^7,8,9,10,11^

## Limitations of the study

The limitation of this study is that the children may not have been accurate in reporting the time for sedentary behaviour and activities in which they participated within the past 7 days of the week because a self-reporting questionnaire was used. Thus, the possibility that errors may have occurred in classifying children’s PA and sedentary behaviours cannot be ruled out. However, the purpose of using a self-report was to obtain a general idea of the time they usually spend on those two activities.
